# Insights to estimate exposure to regulated and non-regulated disinfection by-products in drinking water

**DOI:** 10.1038/s41370-022-00453-6

**Published:** 2022-06-29

**Authors:** Paula E. Redondo-Hasselerharm, Dora Cserbik, Cintia Flores, Maria J. Farré, Josep Sanchís, Jose A. Alcolea, Carles Planas, Josep Caixach, Cristina M. Villanueva

**Affiliations:** 1 ISGlobal, Barcelona, Spain; 2 Universitat Pompeu Fabra (UPF), Barcelona, Spain; 3 CIBER Epidemiología y Salud Pública (CIBERESP), Madrid, Spain; 4 Mass Spectrometry Laboratory/Organic Pollutants, Institute of Environmental Assessment and Water Research, IDAEA-CSIC, Barcelona, Spain; 5 Catalan Institute for Water Research, ICRA, Girona, Spain; 6 University of Girona, Girona, Spain; 7 IMIM (Hospital del Mar Medical Research Institute), Barcelona, Spain

**Keywords:** Drinking water, Disinfection by-products, Exposure assessment, Filtered water, Bottled water, Urine

## Abstract

**Background:**

Knowledge about human exposure and health effects associated with non-routinely monitored disinfection by-products (DBPs) in drinking water is sparse.

**Objective:**

To provide insights to estimate exposure to regulated and non-regulated DBPs in drinking water.

**Methods:**

We collected tap water from homes (*N* = 42), bottled water (*N* = 10), filtered tap water with domestic activated carbon jars (*N* = 6) and reverse osmosis (*N* = 5), and urine (*N* = 39) samples of participants from Barcelona, Spain. We analyzed 11 haloacetic acids (HAAs), 4 trihalomethanes (THMs), 4 haloacetonitriles (HANs), 2 haloketones, chlorate, chlorite, and trichloronitromethane in water and HAAs in urine samples. Personal information on water intake and socio-demographics was ascertained in the study population (*N* = 39) through questionnaires. Statistical models were developed based on THMs as explanatory variables using multivariate linear regression and machine learning techniques to predict non-regulated DBPs.

**Results:**

Chlorate, THMs, HAAs, and HANs were quantified in 98–100% tap water samples with median concentration of 214, 42, 18, and 3.2 μg/L, respectively. Multivariate linear regression models had similar or higher goodness of fit (R2) compared to machine learning models. Multivariate linear models for dichloro-, trichloro-, and bromodichloroacetic acid, dichloroacetonitrile, bromochloroacetonitrile, dibromoacetonitrile, trichloropropnanone, and chlorite showed good predictive ability (*R*^2^ = 0.8–0.9) as 80–90% of total variance could be explained by THM concentrations. Activated carbon filters reduced DBP concentrations to a variable extent (27–80%), and reverse osmosis reduced DBP concentrations ≥98%. Only chlorate was detected in bottled water samples (*N* = 3), with median = 13.0 µg/L. Creatinine-adjusted trichloroacetic acid was the most frequently detected HAA in urine samples (69.2%), and moderately correlated with estimated drinking water intake (*r* = 0.48).

**Significance:**

Findings provide valuable insights for DBP exposure assessment in epidemiological studies. Validation of predictive models in a larger number of samples and replication in different settings is warranted.

**Impact statement:**

Our study focused on assessing and describing the occurrence of several classes of DBPs in drinking water and developing exposure models of good predictive ability for non-regulated DBPs.

## Introduction

Water disinfection is a necessary public health intervention to prevent waterborne infections. However, unintended disinfection by-products (DBPs) are formed during chemical disinfection processes [1]. DBPs occur in complex mixtures, and their relative concentrations depend on the characteristics of organic matter in the raw water, the treatment and disinfectant used, and the length and condition of the distribution system [2–4]. More than 600 DBPs have been identified to date, constituting a widespread exposure in the population worldwide through drinking water consumption, inhalation, and dermal contact [4]. Long-term exposure to DBPs has been consistently associated with increased bladder cancer risk [5]. DBP exposure also has been associated with a number of reproductive and pregnancy outcomes, although evidence is less consistent [4].

The current state of knowledge about the health effects linked to DBP exposure mostly relies on regulated DBPs. The EU currently regulates total trihalomethanes (THMs) and bromate in finished drinking water, although new regulations will be enforced from 2023 to incorporate haloacetic acids (HAAs), chlorite, and chlorate [6]. Epidemiological research on emerging or non-regulated DBPs is limited in a large extent by the lack of adequate routine monitoring data necessary to evaluate exposure in human studies. However, regulated DBPs are a minor fraction of total halogenated DBPs [7], and may not be the primary drivers of toxicity [8]. Epidemiological studies have mainly evaluated trihalomethanes (THMs) and, to a lower extent HAAs [4]. THMs have been typically used as DBP markers for association analyses of human health effects, although one can argue that they might not necessarily be the causal agents [9]. Among the nonvolatile HAAs, trichloroacetic acid (TCAA) received increased attention as a proxy DBP biomarker due to significant correlations reported between TCAA concentrations in urine and ingested TCAA from drinking water [10–13]. However, there is limited knowledge about other urinary HAAs.

A better understanding of the health effects associated with DBP exposure requires the evaluation of a range of DBP classes in addition to THMs [14]. The lack of adequate biomarkers reflecting long-term exposure forces epidemiologists to use water concentrations as the main component of exposure assessment, together with modeling approaches to estimate historical THM concentrations [15]. A number of studies have developed predictive models of THMs based on water parameters [16]. However, the use of models to predict non-regulated DBPs in finished drinking water with exposure assessment purposes has not been explored, to our knowledge.

We aimed to provide insights to estimate exposure to a wide range of DBPs in drinking water in Barcelona (Spain), by (1) describing occurrence in tap and bottled water; (2) developing statistical models to predict non-regulated DBPs based on routinely monitored parameters in the public water supply; (3) evaluating the effect of domestic filters on tap water concentrations; and (4) exploring the use of DBPs in urine as biomarkers of exposure though drinking water. Findings are potentially applicable for exposure assessment in epidemiological studies to evaluate health effects associated with non-regulated DBPs.

## Material and methods

### Study area

Barcelona city and the metropolitan area (North-East Spain) are located in a coastal area in the Mediterranean sea characterized by dry weather, whose main drinking water supply rely on surface sources (Llobregat and Ter rivers). The Llobregat river is severely impacted by anthropogenic activities, and contains a higher bromide concentration (range = 2.5–10 mg/L) compared to the Ter river (range = 0.5–5 mg/L) [17], which leads to the predominance of brominated THMs in drinking water [18]. Although historically high concentrations of total THMs [19] have been dramatically reduced after incorporating membrane-based technology in the drinking water plants, there is still a relative predominance of brominated species [18].

### Study participants and data

We aimed to enroll volunteers living in 42 locations (one per postal code) to represent the geography of Barcelona. Participants were reached through advertisements in social media and were contacted via email. A brief online screening questionnaire including the postal code of residence and type of water consumed was used to create a roster of potential volunteers. We recruited 39 volunteers and conducted home visits to collect urine and tap water samples between August 31st and October 16th of 2020. For 3 postal codes we failed to identify volunteers thus we collected drinking water samples from public fountains during the same period. Among the 39 volunteers, *N* = 11 used domestic filters. Gender balance was also used as a secondary selection criterion, in order to enroll both men and women. Participants provided written consent prior to voluntary participation. Personal information (sociodemographic, anthropometrics, lifestyle) and drinking water consumption habits (source, amount) were collected through a self-administered online questionnaire. We semi-quantitatively ascertained the amount of bottled water, unfiltered tap water, and filtered tap water consumed at home and outside (≤1, 1, 2, 3–4, 5–6, >6 glasses/day, where 1 glass = 250 mL). The study was approved by the Parc de Salut Mar Ethics committee.

### Sample collection

#### Tap water samples

We collected unfiltered tap water samples at 42 locations, plus filtered tap water samples in a subset of 11 homes: *N* = 6 activated carbon (pitcher type), *N* = 5 reverse osmosis filters. Tap water samples (both unfiltered and filtered) were collected in 4 containers: (1) 2.5 L glass bottle for HAAs analysis; (2) 500 mL glass bottle for chlorate and chlorite analysis; (3) 250 mL glass bottle for THMs, haloacetonitriles (HANs), haloketones (HKs), and trichloronitromethane (TCNM) analysis; and (4) 1 L glass bottle for physicochemical parameters analysis. Ascorbic acid was added as quenching agent prior to the collection of the water samples in bottles aimed at quantifying HAAs, THMs, HANs, HKs, and TCNM. Tap water samples were collected after leaving cold water running for 2 min approximately. Bottles without quencher were rinsed twice with tap water on site. Bottles with quencher were slowly filled to the top to avoid air bubbles, an air chamber and quencher loss, and were finally gently shaken for at least 30 s. Samples were transported in a portable cooler with ice packs to the research center, where samples were stored in the refrigerator (≈4 °C) until shipment to the laboratories within 1–4 days.

#### Bottled water samples

We included samples from 10 brands of natural mineral water selected among the most popular in the area. We purchased 1.5 L polyethylene terephthalate (PET) bottles at local supermarkets, that were transported at room temperature to the laboratory.

#### Urine samples

First morning-void spot urine samples were collected from 39 volunteers, on the same day that the tap water samples were collected. Participants received the container in advance together with written instructions to self-collect urine samples on the day of the home visit. Urine samples were collected in a 70-mL sterile plastic container and were placed in the fridge until the visit of study personnel. Urine samples were transported at ≈4 °C to the research center and stored at −20 °C until the analysis at the end of enrollment.

### Laboratory analyses

Details about analytical methods are in the Supplementary Information (SI). Analytical methodologies and limits of quantification (LOQ) and detection (LOD) are summarized in Table S1 for the different analytes in drinking water and urine. LOQs of DBPs in water ranged between 0.1 µg/L (THMs, HANs, HKs, trichloronitromethane) and 10 µg/L (chlorate, chlorite), and LODs of HAAs in urine were in the range between 0.02 µg/L (TCAA) and 3.98 µg/L (iodoacetic acid) (Table S1). Drinking water samples were analyzed for 11 HAAs, 4 THMs, 4 HANs, 2 HKs, TCNM, chlorate and chlorite. Chlorate and chlorite were measured directly and HAAs were pre-concentrated by online solid phase extraction (SPE). HAAs, chlorite and chlorate were analyzed by tandem mass spectrometry coupled to liquid chromatography (LC–MS/MS). Specifically, HAAs were analyzed according to the method developed by Planas *et al*. with some modifications [20]. Analysis of THMs, HANs, HKs, and TCNM were performed by liquid-liquid salted microextraction and gas chromatography (GC Trace 1300, Thermo Fisher Scientific) coupled to a mass spectrometer (GC–MS/MS, Thermo Fisher Scientific).

Urine samples were only analyzed for 11 HAAs with the aim to examine their biomarker potential for exposure assessment in epidemiological studies. HAAs were analyzed using off-line SPE and LC–MS/MS based on the methods previously developed [21, 22]. Urinary creatinine was determined using an automated alkaline picrate method [23]. The limit of detection was 2.9 mg/dL. We divided the concentrations of HAAs in urine samples by the creatinine concentrations to adjust for the urinary concentration (reported as μg/g creatinine).

For all LC–MS/MS analyses, a TSQ quantum triple quadrupole mass spectrometer equipped with an electrospray ionization (ESI) source (Thermo Fisher Scientific, San Jose, CA, USA), a Finnigan Surveyor MS plus pump and a HTC PAL autosampler were used. The analyses were carried out in negative ion electrospray and multiple reaction monitoring acquisition mode (MRM). The spray voltage was chosen at 3.0 kV and the tube lens voltage and collision energy were optimized for each m/z and for each transition, respectively. The ion transfer tube temperature was set at 250 °C. Nitrogen was used as a sheath and auxiliary gas at flow rates of 65 psi and 15 arbitrary units (a.u.), respectively. The argon gas collision-induced dissociation was used with a pressure of 1.5 millitorr (mTorr). Data acquisition was performed with Xcalibur 2.0.7 software (Thermo Fisher Scientific).

Quantification and quality control measures to comply with the 2002/657/EC Commission Decision [24] are described in detail in the SI. All chemicals were measured in all drinking water types, except for THMs, HANs, HKs and TCNMs, which were not analyzed in bottled water because of the low THM levels detected in bottled water in a previous study [25]. More information about the analytical procedure including physicochemical parameters and reagents are detailed in the SI.

### Statistical analysis

#### Descriptive analyses

Maximum, percentiles, mean, and standard deviation (SD) were calculated for measurements >LOQ. The bromine incorporation factor (BIF) was calculated for THMs (1) and HAAs (dihalogenated species (DXAAs) (2) and trihalogenated (TXAAs) (3)) to assess the molar contribution of the brominated species with the following equations (details provided in the SI):

1. BIF(THMs) = (0 × [TCM] + 1 × [BDCM] + 2 × [DBCM] + 3 × [TBM])/([TCM] + [BDCM] + [DBCM] + [TBM])
2. BIF(DXAA) = (0 × [DCAA] + 1 × [BCAA] + 2 × [DBAA])/([DCAA] + [BCAA] + [DBAA])
3. BIF(TXAA) = (0 × [TCAA] + 1 × [BDCAA] + 2 × [DBCAA] + 3 × [TBAA])/([TCAA] + [BDCAA] + [DBCAA] + [TBAA])

Normalized BIF was calculated by dividing BIF by the number of halogen substituents. Spearman rank correlation coefficients were calculated to evaluate the degree of correlation between individual DBPs as well as between ingested TCAA and urine levels. A principal component analysis (PCA) was performed to describe and reduce the dimensionality of the different DBP classes. Samples (water, urine) with concentrations <LOQ were assigned LOQ/2 to estimate correlations and the PCA.

#### Multivariate predictive models

We used linear regression and machine learning to develop models predicting non-regulated DBPs based on routine monitoring parameters. Linear regression models were based on 4 THM species (trichloromethane: TCM; bromodichloromethane: BDCM; dibromochloromethane: DBCM; and bromoform: TBM) as independent variables. Conductivity was not considered due to its high correlation with THMs. For each DBP and each transformation of the independent variables (no transformation, log, square root, squared) we performed 15 variations of linear regression models within the possible combinations of independent variables (4 simple models, 11 multiple models). We selected the best model for each DBP and each transformation based on the highest R-squared (R^2^) and variance inflation factor (VIF) lower than 10 to avoid multicollinearity. As a next step, we used 5-fold cross validation as a method to estimate the prediction accuracy of these models and selected the final linear models based on the highest coefficient of determination (*R*^2^), narrower confidence interval (95% CI) and lower Root Mean Squared Error (RMSE) for each DBP.

Super learner (SL) modeling is a machine learning method and prediction technique that combines several individual predictive algorithms (library of algorithms) into a new individual model: a weighted combination (ensemble). Separate models were built to predict DBPs concentrations using fivefold cross-validated SL based on the 4 THMs, conductivity, pH, and geocodes as explanatory variables. SL modeling was developed with 3 different cross-validated models using different individual algorithms: Model 1 = algorithm library including generalized linear model, Bayesian GLM, random forest (from ‘random forest’ and ‘ranger’ packages), multivariate adaptive regression splines, local polynomial regression, neural network, adaptive polynomial splines; Model 2 = same as Model 1 plus Random Forest algorithm modification; Model 3 = same as Model 2 plus additional screening algorithms for the input variables. For each DBP, models with the highest R^2^, narrower 95% CI and lower RMSE were selected for comparisons with linear regression models.

All statistical analyses were performed using R version 4.1.1 (2021-08-10) [26]. Packages ggplot2, ggpubr, factoextra, RVAideMemoire, *Superlearner (*v 2.0-28) [27], *caret* (v 6.0-88) [28] were used.

#### Effect of domestic filters on DBPs concentrations in tap water

Average concentrations before and after filtration were compared using paired t-tests, after checking the normality of the resulting difference with the Shapiro–Wilk test. Log or square root transformation was necessary for some of the variables to meet the assumption of normality. The homogeneity of the variances was evaluated for each variable and considered in the paired t-test. The average percentage change was calculated as the after-before difference in the concentration relative to the average concentration before filtration.

#### Estimated DBP ingestion

We identified the primary source of drinking water at home and estimated residential DBP exposure by multiplying the volume (in liters) by the concentration of DBPs in the specific type of water consumed.

## Results

Characteristics of the study population is presented in Table 1. Twenty-four participants (60.5%) were female, 14 (36.8%) were male and 1 (2.6%) was non-binary. Mean age and body mass index in the study population were, respectively, 41 years old and 22.7 kg/m^2^. Unfiltered tap water was the drinking water type with the highest mean volume consumed (0.6 L/day) at home, followed by bottled (0.5 L/day) and filtered tap water (0.4 L/day). On average, participants spent 9.2 min/day showering, and 4 participants reported to regularly swim in chlorinated pools.

**Table 1:** Characteristics of the study population (*N* = 39)^a^.

Variable	Mean ± SD (range)
Age, years	40.7 ± 10.2 (26–76)
Body mass index, kg/m^2^	22.7 ± 2.9 (18.6–30.1)
Drinking water consumption (home), L/day	
Tap	0.6 ± 0.5 (0.1–1.5)
Filtered	0.4 ± 0.5 (0.1–1.5)
Bottled	0.5 ± 0.4 (0.3–1.5)
Showering time, minutes/day	9.2 ± 4.7 (2–30)
	***N*** **(%)**
Gender	
Male	14 (36.8)
Female	24 (60.5)
Other (non-binary)	1 (2.6)
Education	
High school	4 (10.3)
≥University	35 (89.7)
Swimming in chlorinated pool	
Yes	4 (10.3)
No	35 (89.7)
Smoking^b^	
Yes	4 (10.3)
No	35 (89.7)

^a^1 missing value in body mass index, 2 missing showering time values.

^b^At least 1 cigarette/day or 1 cigar/week in the last 6 months.

### DBP occurrence in tap and bottled water

Table 2 shows the DBP concentrations in tap water samples, and physicochemical parameters are provided in Table S2. THMs and HANs were present in all unfiltered tap water samples. Specifically, BDCM, DBCM, TBM, bromochloroacetonitrile (BCAN) and dibromoacetonitrile (DBAN), were quantified in at least 90% of samples. HAAs were detected in 98% of the samples, being monobromo-, dibromo-, bromodichloro-, and trichloro- acetic acids (MBAA, DBAA, BCAA, TCAA) quantified in more than 48% of the samples. Chlorate, chlorite, and trichloropropanone (TCP) were found in 98, 62 and 36% of the tap water samples, respectively. Six out of the 24 DBPs analyzed were below the LOQ in all samples: monochloro- (MCAA), monoiodo- (MIAA), and diiodo- (DIAA) acetic acid, trichloroacetonitrile (TCAN), dichloropropanone (DCP) and trichloronitromethane (TCNM). The median value of total THMs, HAAs and HANs, TCP, chlorite and chlorate in tap water, calculated using values >LOQ, were 42, 18, 3.2, 1.2, 53.9 and 214 μg/L, respectively (Table 2).

**Table 2:** Occurrence and concentrations (µg/L) of disinfection by-products (DBPs) in tap water samples (*N* = 42) above the limit of quantification (LOQ).

	*N* ≥ LOQ	% ≥ LOQ	Min	Perc25	Perc50	Perc75	Max	Mean	SD
Haloacetic acids (HAAs)									
Monochloroacetic acid (MCAA)	0	0	<2.0	<2.0	<2.0	<2.0	<2.0	<2.0	–
Dichloroacetic acid (DCAA)	17	41	<0.5	6.4	9.8	12	15	9.2	3.9
Trichloroacetic acid (TCAA)	20	48	<0.5	12	18	22	24	15	7.9
Monobromoacetic acid (MBAA)	24	57	<0.5	0.8	1.0	1.2	1.7	1.0	0.3
Dibromoacetic acid (DBAA)	28	67	<0.5	3.8	7.4	8.6	14	6.3	3.5
Tribromoacetic acid (TBAA)	18	43	<0.5	2.0	2.9	3.9	6.6	3.0	1.6
Bromochloroacetic acid (BCAA)	26	62	<0.5	1.0	1.7	2.1	4.9	1.7	0.9
Dichlorobromoacetic acid (BDCAA)	16	38	<0.5	1.6	1.8	2.3	3.7	1.9	0.7
Dibromochloroacetic acid (DBCAA)	17	41	<0.5	1.0	1.3	2.0	3.4	1.6	0.8
Iodoacetic acid (IAA)	0	0	<0.5	<0.5	<0.5	<0.5	<0.5	<0.5	–
Diiodoacetic acid (DIAA)	0	0	<0.5	<0.5	<0.5	<0.5	<0.5	<0.5	–
Total HAAs	41	98	<LOQ	11	18	30	39	20	12
Trihalomethanes (THMs)									
Chloroform (TCM)	27	64	0.3	1.2	23	28	36	17	14
Bromodichloromethane (BDCM)	42	100	0.3	1.4	2.4	8.6	12	4.6	3.7
Dibromochloromethane (DBCM)	42	100	1.7	3.3	8.0	11	27	8.5	5.7
Bromoform (TBM)	38	91	0.2	12	25	29	58	23	15
Total THMs	42	100	17	38	42	49	83	45	13
Haloacetonitriles (HANs)									
Dichloroacetonitrile (DCAN)	23	55	0.2	0.5	1.7	2.1	2.6	1.4	0.8
Bromochloroacetonitrile (BCAN)	42	100	0.1	0.4	0.5	0.6	0.9	0.5	0.2
Dibromoacetonitrile (DBAN)	38	91	0.1	0.9	2.7	3.2	4.3	2.2	1.4
Trichloroacetonitrile (TCAN)	0	0	<0.1	<0.1	<0.1	<0.1	<0.1	<0.1	–
Total HANs	42	100	1.3	2.6	3.2	3.9	5.0	3.3	0.9
Haloketones (HKs)									
1,1,1-Trichloropropanone (TCP)	15	36	<0.1	1.1	1.2	1.3	2.0	1.2	0.4
1,1-Dichloropropanone (DCP)	0	0	<0.1	<0.1	<0.1	<0.1	<0.1	<0.1	–
Other DBPs									
Trichloronitromethane (TCNM)	0	0	<0.1	<0.1	<0.1	<0.1	<0.1	<0.1	–
Chlorite	26	62	<10.0	16.9	53.9	113	149	61.8	49.8
Chlorate	41	98	<10.0	159	214	296	367	225	79.9

Median BIF values for THMs, dihalogenated (DXAA) and trihalogenated (TXAA) HAAs were 2.48, 1.78 and 0.08, respectively. Normalized median BIF values were, respectively, 0.83, 0.89 and 0.03. Samples with lower bromide substitution (TCM > TBM) had similar concentrations of total THMs and HAAs, while samples with higher bromide substitution (TBM > TCM), generally showed higher THM concentrations compared to HAAs (Table S3, Fig. S1). Principal component analysis revealed two components of DBPs: (1) chlorinated species (DCAA, TCAA, BDCAA, TCM, BDCM, DCAN, DBAN, chlorite, TCP) and TBM, explaining 61.5% of the total variance; and (2) dominated by brominated species (BCAN, DBCAA, TBAA) and chlorate, that explained 16.3% of the total variance (Table S4, Fig. S2).

Spearman correlation coefficients (*ρ*) between DBPs are shown in Table S5. Highest *ρ* (≥0.90) were found for TBM-DBAN (*ρ* = 0.93), DBCM-DBAN (ρ = 0.91), TCAA-DCAN (*ρ* = 0.91), TCM-TCAA (*ρ* = 0.91), DCAA-BDCAA (*ρ* = 0.90). Total THMs vs. total HAAs were weakly correlated (*ρ* = 0.32), and correlations in absolute value (|*ρ*|) between individual THMs and HAAs ranged from 0.12 to 0.91, in opposite directions at times. Individual THMs were correlated with individual HANs to a variable extent (range |*ρ*|= 0.15–0.93), and total THMs vs. total HANs correlation was *ρ* = 0.59. Individual THMs significantly correlated with TCP and chlorite (range |*ρ*| = 0.48–0.76), in opposite directions in some cases. Individual HAAs were not correlated with BCAA (*ρ* ≤ 0.34), and correlation (|ρ|) with DCAN and DBAN ranged, respectively, 0.40–0.91 and 0.22–0.79 in varying in directions. Chlorate showed weak correlations (*ρ* ≤ 0.44) except for a moderate correlation with chlorite (*ρ* = 0.58).

Table S6 presents correlation coefficients between physicochemical parameters and DBPs. Conductivity was positively correlated with hardness (*ρ* = 0.86) and negatively with TOC (*ρ* = −0.83). Conductivity was negatively correlated with total HAAs (*ρ* = −0.82), total THMs (*ρ* = −0.39), DCAN (*ρ* = −0.78), BCAN (ρ = −0.41), TCP (*ρ* = −0.75), chlorite (*ρ* = −0.70), chlorate (*ρ*= −0.48); and positively correlated with the BIF of THMs (*ρ* = 0.82), BIF of DXAA, (*ρ* = 0.66), and TXAA (*ρ* = 0.65). Moreover, individual and total DBPs were moderate to strongly correlated with hardness, TOC, and pH, in opposite directions in some cases.

Results of bottled water brands showed that only chlorate was quantified, in three out of ten samples (median = 13.0 µg/L, IQR = 12.4–22.4 µg/L). A summary of the physicochemical parameters measured in bottled water is given in Table S2.

### Multivariate predictive models

Table 3 summarizes the 5-fold cross-validated model parameters of linear regression and super learner models for 14 individual DBPs, total brominated HAAs, total chlorinated HAAs, total HAAs and total HANs. Models for DCAA, TCAA, BDCAA, total HAAs, total brominated HAAs, total chlorinated HAAs, regulated HAAs, DCAN, BCAN, DBAN, total HANs, TCP, and chlorite had cross-validated *R*^2^ > 0.7 and lower 95% LCI > 0.5, showing an acceptable predictive capacity. However, models for other haloacetic acids (MBAA, DBAA, TBAA, BCAA, DBCAA) and chlorate showed poor goodness of fit (cross-validated *R*^2^ < 0.7, LCI < 0.5).

**Table 3:** Cross-validated (fivefold) linear regression and super learner models for non-regulated disinfection by-products (DBPs) based on routinely monitored parameters as explanatory variables.

	Linear regression				Super learner model		
Analyte	Independent variable(s)	Transformation^a^	*R*^2^ (95% CI)	RMSE(SD)	Model^b^	*R*^2^ (95% CI)	RMSE (SD)
**Haloacetic acids (HAAs)**							
**DCAA**	**TCM**	–	**0.89 (0.79, 0.95)**	2.17 (1.08)	1	**0.82 (0.59, 0.92**)	2.02 (1.28)
**TCAA**	**TCM**	–	**0.97 (0.94, 0.98)**	2.12 (0.61)	3	**0.97 (0.96, 0.98**)	1.41 (0.36)
MBAA	BDCM,DBCM	–	0.50 (0.14, 0.70)	0.37 (0.06)	2	0.43 (0.11, 0.63)	0.32 (0.07)
DBAA	TCM,BDCM,DBCM	log	0.65 (0.33, 0.81)	2.45 (0.78)	3	0.64 (0.43, 0.77)	2.36 (1.31)
TBAA	TCM,BDCM,DBCM	log	0.43 (0.03, 0.66)	1.35 (0.26)	1	0.19 (−0.12, 0.41)	1.48 (0.40)
BCAA	BDCM	–	0.35 (0.03, 0.57)	0.92 (0.25)	3	0.19 (0.01, 0.34)	0.89 (0.29)
**BDCAA**	**TCM**	–-	**0.82 (0.32, 0.95)**	0.38 (0.27)	2	0.64 (0.32, 0.80)	0.50 (0.32)
DBCAA	DBCM,TBM	sqrt	0.44 (−0.09, 0.71)	0.72 (0.21)	3	0.09 (−0.31, 0.38)	0.78 (0.22)
**Brominated HAAs**^c^	**TCM,BDCM,DBCM**	log	**0.72 (0.50, 0.85)**	3.41 (1.20)	1	0.58 (0.37, 0.72)	3.67 (0.43)
**Chlorinated HAAs**^d^	**TCM**	–	**0.96 (0.92, 0.97)**	3.20 (0.65)	3	**0.94 (0.90, 0.97)**	2.94 (1.76)
**Total HAAs**	**TCM,BDCM,DBCM,TBM**	log	**0.85 (0.77, 0.90)**	5.87 (0.95)	1	**0.77 (0.65, 0.85)**	5.83 (0.99)
**Haloacetonitriles (HANs)**							
**DCAN**	**BDCM,DBCM**	sqrt	**0.97 (0.95, 0.98)**	0.20 (0.07)	1	**0.95 (0.92, 0.97)**	0.20 (0.08)
**BCAN**	**BDCM,DBCM**	sqrt	**0.76 (0.60, 0.86)**	0.09 (0.01)	3	0.63 (0.40, 0.78)	0.10 (0.03)
**DBAN**	**TCM,BDCM,DBCM,TBM**	log	**0.89 (0.83, 0.93)**	0.53 (0.18)	3	**0.92 (0.86, 0.95)**	0.41 (0.14)
Total HANs	BDCM,DBCM	sqrt	0.65, (0.44, 0.78)	0.61 (0.12)	3	0.58 (0.38, 0.71)	0.57 (0.15)
**Haloketones (HKs)**							
**TCP**	**TCM,BDCM,DBCM,TBM**	log	**0.82 (0.70, 0.89)**	0.29 (0.13)	1	**0.81 (0.48, 0.93)**	0.26 (0.15)
**Other DBPs**							
Chlorate	DBCM,TBM	sqrt	0.40 (−0.081, 0.74)	71.25 (19.96)	2	0.34 (−0.05, 0.58)	65.95 (21.11)
**Chlorite**	**BDCM,TBM**	sqrt	**0.77 (0.60, 0.86)**	25.49 (12.1)	2	**0.72 (0.45, 0.86)**	23.63 (12.15)

Models were built using the total number of samples (*N* = 42) after converting values <LOQ into LOQ/2. Bold indicates models with *R*^2^ > 0.7 and lower 95% confidence interval > 0.5. All parameters in this table are fivefold cross-validated.

*RMSE* root mean squared error, *DCAA* dichloroacetic acid, *TCM* trichloromethane, *TCAA* trichloroacetic acid, *MBAA* monobromoacetic acid, *BDCM* bromodichloromethane, *DBCM* dibromochloromethane, *DBAA* dibromoacetic acid, T*BAA* tribromoacetic acid, *BCAA* bromochloroacetic acid, *BDCAA* bromodichloroacetic acid, *DBCAA* dibromochloroacetic acid, *DCAN* dichloroacetonitrile, *BCAN* bromochloroacetonitrile, *DBAN* dibromoacetonitrile, *TBM* bromoform, TCP 1,1,1-trichloropropanone.

^a^Transformation of independent variables.

^b^Model 1= algorithm library including generalized linear model, Bayesian GLM, random forest, multivariate adaptive regression splines, local polynomial regression, neural network, adaptive polynomial splines; Model 2 = same as Model 1 plus Random Forest algorithm modification; Model 3 = same as Model 2 plus additional screening algorithms for the input variables.

^c^Brominated HAAs include MBAA, DBAA, and TBAA.

^d^Chlorinated HAAs include DCAA, TCAA, BCAA, BDCAA, and DBCAA.

### Effect of domestic filters on DBPs concentrations in tap water

Activated carbon filters significantly reduced average total HAAs concentrations (−52%, *p* value = 0.045), total THMs (−80%, *p* value = 0.003), and total HANs (−75%, *p* value = 0.001) (Table 4). Average TCP, chlorite and chlorate concentrations were reduced −63%, −60% and −27% (*p* value > 0.05, respectively) after activated carbon filtration, although differences did not reach statistical significance. Activated carbon filters significantly reduced free chlorine from 1.9 to 0.1 mg/L (*p* value = 0.01) and total chlorine from 2.4 to 0.2 mg/L (*p* value = 0.001) but did not reduce total organic carbon (Table S7). Reverse osmosis filters reduced total HAAs, total HANs (*p* value < 0.001), TCP and chlorite concentrations to levels <LOQ (−100%) and reduced THMs and chlorate levels −99% and −98%, respectively (Table 4). Reverse osmosis significantly reduced total organic carbon, conductivity, hardness, and TOC (*p* value < 0.001) (Table S7).

**Table 4:** Effect of domestic filters on disinfection by-products (DBPs) concentrations in tap water.

	N ≥ LOQ	% ≥ LOQ	Min	Perc25	Perc50	Perc75	Max	Mean	SD	Average % Change
Total haloacetic acids (HAAs)										
Before AC filter	6	100	2.7	14	16	32	38	21	14	−52
After AC filter	6	100	1.5	5.2	11	15	16	9.9	6.3	
Before RO filter	4	80	<LOQ	19	20	20	21	19	2.4	−100
After RO filter	0	0	<LOQ	<LOQ	<LOQ	<LOQ	<LOQ	<LOQ	–	
Total trihalomethanes (THMs)										
Before AC filter	6	100	34	44	51	56	82	53	16	−80
After AC filter	6	100	0.1	6.1	11	16	20	11	7.4	
Before RO filter	5	100	28	35	39	46	48	39	8.0	−99
After RO filter	3	60	<LOQ	0.1	0.1	0.8	1.5	0.6	0.8	
Total haloacetonitriles (HANs)										
Before AC filter	6	100	3.2	3.3	3.5	3.7	4.7	3.6	0.6	−75
After AC filter	5	83	<LOQ	0.4	0.7	1.2	1.8	0.9	0.6	
Before RO filter	5	100	2.2	2.7	3.9	4.0	4.2	3.4	0.9	−100
After RO filter	0	0	<LOQ	<LOQ	<LOQ	<LOQ	<LOQ	<LOQ	–	
1,1,1-trichloropropanone (TCP)										
Before AC filter	3	50	<0.01	0.8	1.1	1.1	1.2	0.9	0.4	−63
After AC filter	1	17	<0.01	0.3	0.3	0.3	0.3	0.3	0	
Before RO filter	1	17	<0.01	1.3	1.3	1.3	1.3	1.3	0	−100
After RO filter	0	0	<0.01	<0.01	<0.01	<0.01	<0.01	<0.01	–	
Chlorite										
Before AC filter	5	83	<10	16.8	17.2	119	142	61.2	63.8	−60
After AC filter	3	50	<10	17.0	17.0	28.4	39.7	24.5	13.1	
Before RO filter	2	40	<10	22.6	22.6	22.6	22.6	22.6	0	−100
After RO filter	0	0	<10	<10	<10	<10	<10	<10	–	
Chlorate										
Before AC filter	6	100	153	193	219	314	367	248	86.3	−27
After AC filter	6	100	116	144	178	185	296	181	62.9	
Before RO filter	4	80	<10.0	167	183	232	341	217	84.4	−98
After RO filter	5	100	18.2	28.1	34.2	35.5	44.4	32.1	9.70	

Number of samples (*N*, %) with DBPs above the limit of quantification (LOQ) and concentrations (µg/L) in tap water before and after activated carbon (AC, *N* = 6) and reverse osmosis (RO, *N* = 5) filters.

### Urinary biomarkers of exposure

**Fig. 1:**
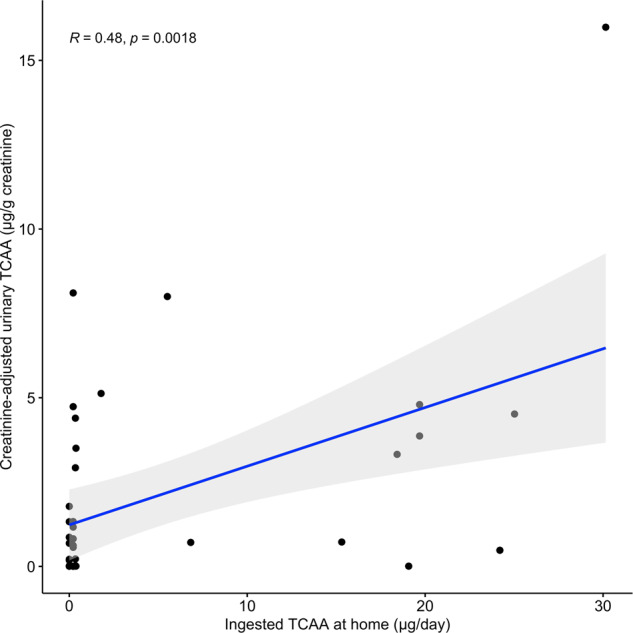
Creatinine-adjusted urinary TCAA (µg/g creatinine) vs. TCAA ingestion from home drinking water (µg/day). Ingested TCAA from drinking water at home was calculated with reported individual tap water consumption (non-filtered, filtered or bottled respectively (L/day)). TCAA concentrations <LOD were assigned LOD/2.

Table 5 presents summary statistics for HAAs concentration in urine. Urine samples (*N* = 39) had detectable levels of 5/11 HAAs above LOD with TCAA being the most prevalent (69.2% detection rate overall, 50% among bottled water users, and 40% among RO users), followed by DBCAA (23.1%), BDCAA (20.5%), DCAA (10.3%), MCAA (2.6%). Other HAAs were below LOD (MBAA, DBAA, IAA, DIAA). No results are shown for BCAA and TBAA due to the great matrix effect and instability, respectively. Urinary TCAA concentrations ranged from non-detectable (<0.02 µg/L) to 33 µg/L (mean = 4.2, median=1.3 µg/L) and from non-detectable (<0.01 µg/g) to 16.0 µg/g (mean = 3.0, median=1.3) after adjusting for creatinine. When considering creatinine-adjusted urinary TCAA measurements below the limit of detection (<LOD; 30.8%), we assigned LOD/2, that resulted in a lower level of adjusted mean urinary TCAA (2.1 µg/g) (Fig. 1). Spearman’s correlation between creatinine-adjusted urinary TCAA and ingested TCAA at home from drinking water was moderate but statistically significant (ρ=0.48, p-value=0.002), explaining approximately 50% of total variability in urinary TCAA (Fig. 1).

**Table 5:** Urinary concentrations of trichloroacetic acid (TCAA) and other haloacetic acids (HAAs) among the study population (*n* = 39).

Analyte	Mean (SD)	Min–Max	Median (IQR)^a^	*N* ≥ LOD (%)
a) Urinary TCAA (µg/L)	4.2 (7.1)	<0.02 to 33.0	1.3 (0.5, 5.3)	27 (69.2%)
b) Creatinine-adjusted urinary TCAA (µg/g)	3.0 (3.5)	<0.01 to 16.0	1.3 (0.7, 4.5)	27 (69.2%)
c) Other Creatinine-adjusted urinary DBPs				
MCAA (µg/g creatinine)	117.2	<0.48 to 117.2	117.2	1 (2.6%)
DCAA (µg/g creatinine)	154.7 (263.3)	<0.3 to 549.6	25.8 (22.5, 158.0)	4 (10.3%)
BDCAA (µg/g creatinine)	23.2 (6.2)	<0.02 to 36.9	20.9 (19.2, 24.6)	8 (20.5%)
DBCAA (µg/g creatinine)	113.0 (52.5)	<0.6 to 240.9	92.4 (91.0, 116.0)	9 (23.1%)
MBAA (µg/g creatinine)	<LOD	<LOD	<LOD	0.0
DBAA (µg/g creatinine)	<LOD	<LOD	<LOD	0.0
IAA (µg/g creatinine)	<LOD	<LOD	<LOD	0.0
DIAA (µg/g creatinine)	<LOD	<LOD	<LOD	0.0
d) Estimated TCAA concentration in drinking water consumed at home (µg/L)				
Tap	15.8 (7.8)	<0.5–23.9	18.2 (14.9, 21.8)	19 (48.7%)
Filtered tap	7.1 (1.1)	<0.5–7.8	7.1 (6.7, 7.4)	2 (5.1%)
Bottled	0.0	0.0	0.0	0.0
e) Estimated ingested TCAA from drinking water at home (µg/day)^b^	4.9 (8.9)	0–30.2	0.2 (0.0, 3.7)	39 (100%)

*N* and % and descriptive statistics based on samples >LOD.

*TCAA* trichloroacetic acid, *MCAA* monochloroacetic acid, *DCAA* dichloroacetic acid, *BDCAA* bromodichloroacetic acid, *DBCAA* dibromochloroacetic acid, *MBBA* monobromoacetic acid, *MBAA* monobromoacetic acid, *DBAA* dibromoacetic acid, *IAA* iodoacetic acid, *DIAA* diiodoacetic acid.

^a^Interquartile range is the 25th–75th percentiles.

^b^Calculated with reported individual tap water consumption (questionnaire): non-filtered, filtered or bottled respectively (L/day). TCAA drinking water concentrations below <LOD were assigned LOD/2.

## Discussion

### DBP occurrence in tap and bottled water

In the present study, a wide range of DBPs were analyzed in drinking water (tap and bottled). Unfiltered tap water is the primary source of human exposure to these chemicals. The patterns of occurrence indicate that although both brominated and chlorinated DBPs were present, brominated species were found in a larger number of samples. Results are in line with previous studies in the study area, that reported higher levels of brominated compared to chlorinated THMs and HAAs in the tap water of Barcelona [3, 29]. Moreover, our results of high brominated DBPs and THM concentrations are consistent with previous studies that found higher bromide concentrations in water to cause the formation of mainly brominated THMs and reduced formation of HAAs [30]. These results are of high importance, because brominated DBPs are reportedly more cytotoxic and genotoxic than chlorinated species and therefore there is a need to minimize the formation of brominated DBPs [1].

The median THM (42 µg/L) and HAA (18 µg/L) levels in this study compared to a study conducted in 2010 (median THM = 85 µg/L, median HAA∼35 µg/L, respectively) suggest that concentrations of these two DBP classes halved in Barcelona [29]. This can be explained by the technological improvement of the Llobregat drinking water treatment plants, which provides ~50% of the drinking water supply for Barcelona [31, 32]. Our study shows that current levels of total THMs and HAAs in the tap water of Barcelona are below the new parametric values set by EU Drinking Water Directive (DWD) (2020/2184) for total THMs (100 µg/L) and 5 HAAs (60 µg/L). These parametric values will be implemented by 2023 into national legislation of EU member states and will be legally binding [6]. Similar regulatory limits were set by the U.S. EPA for total maximum concentrations of 5 HAAs (MCAA, DCAA, TCAA, MBAA, DBAA) < 60 µg/L and <80 µg/L for total THM concentrations in drinking water [33].

Chlorite and chlorate will also be regulated under the new EU directive with a maximum contaminant level of 250 µg/L (or 700 µg/L where a disinfection method that generates chlorite or chlorate is used). Approximately 25% of the tap water samples in our study contained chlorate levels exceeding 250 µg/L (Table 2). Given that the treatment plants use chlorine dioxide, concentrations are below the 700 µg/L legal threshold applying in this case. Chlorate has been found to cause in vitro mutagenic effects and to induce thyroid tumors in male rats [1, 34]. Although adverse human health effects of chlorate have been scarcely investigated, chlorate levels in drinking water have been associated with a higher risk of obstructive urinary defects, cleft palate and spina bifida in newborns [35]. Chlorate is very persistent and previous studies highlight that only reverse osmosis has been recognized to effectively remove it from drinking water [36]. On the other hand, chlorate was detected in three out of ten analyzed samples (mean = 18.9 µg/L) of popular Spanish bottled water samples. Our results showed that chlorate levels in bottled water were approximately one order of magnitude lower than in tap water samples. Other studies reported higher detection rates but lower concentrations of chlorate in bottled water, for instance, in 71.4% (15/21) of samples from the U.S. (min = 0.2 µg/L, max= 5.8 µg/L) [37] and in 90% (9/10) of samples from Japan (mean = 14 µg/L) [38].

Finally, we assessed correlations between DBPs that were the building blocks of the multivariate analysis. Although general patterns were not identified, correlations tended to be stronger and positive between compounds with a similar proportion of equivalent halogenated (chlorine/bromine) substituents, which is consistent with correlations observed in a previous study by Villanueva et al. [3]. Chlorate was the DBP that correlated the weakest with other DBPs, except with chlorite, showing an independent behavior from THMs, HAAs, HANs, and TCP, difficult to predict. Individual THMs were moderate to strongly correlated with other individual DBPs. Specifically, at least one individual THM showed significant positive correlations with individual DBPs of other classes except for chlorate. These results are in line with previous studies that reported strong correlations between THMs and HAAs [39, 40] as well as between THMs and HANs [41, 42]. Our results of correlation analyses went beyond previous studies showing high correlations between specific THMs and other DBPs (TCP, chlorite). Results suggested that total THM levels can be a good indicator for levels of other DBPs depending on the right combination of compounds. This finding was the basis for our multivariate models that aimed to develop predictive models for unregulated DBPs using individual or multiple THMs levels. Moreover, statistically significant strong correlations between DBPs and physicochemical parameters may suggest that conductivity, hardness, TOC and pH are important determinants in the formation of specific DBPs, and we can only speculate that these correlations might as well explain differences in the formation of DBPs among waters of different regions.

### Multivariate predictive models

We developed linear regression and super learner models to predict 14 individual unregulated DBPs based on the routinely monitored THMs. Models for dichloro-, trichloro-, and bromodichloroacetic acid, dichloroacetonitrile, bromochloroacetonitrile, dibromoacetonitrile, trichloropropnanone, and chlorite showed good predictive ability (*R*^2^ = 0.8–0.9) as 80–90% of total variance could be explained by THM concentrations. In contrast, models had *R*^2^ < 0.7, LCI < 0.5 for the remainder DBPs suggesting that these compounds cannot be reasonably predicted based on routine monitoring data.

When comparing models (LM vs. SL), most target compounds (9/18) had a better fit by linear models and 2/18 by super learner models, while 7/18 showed low goodness of fit (*R*^2^ < 0.7; LCI < 0.5). Our results suggest that SL models perform better when predicting TCAA and DBAN. Notably, our study is restricted to data of low dimensionality, but in high-dimensional data, it is proved theoretically that SL will asymptotically outperform LM, since the LM is included in the library of SL algorithms [27, 43].

For HAAs, 3/8 individual compounds (DCAA, TCAA, BDCAA) were based on TCM as main explanatory variable, similarly to total chlorinated HAAs, while total brominated HAAs and total HAAs were better explained by multiple THMs. HANs were better predicted by BDCM & DBCM, and other non-regulated DBPs were predicted by various combinations of THMs. Previous studies aimed to predict THMs and HAAs [44, 45], however less emphasis was placed on individual compounds [16]. Our results go beyond these studies demonstrating the potential to predict a number of individual as well as group-wise concentrations of DBPs based on THMs. Predictive models of DBPs based on routinely monitored parameters are highly applicable in epidemiological research in order to evaluate exposure to non-monitored DBPs using existing records of THMs and other routinely monitored parameters. Although some of the compounds that we considered unregulated, they will be routinely monitored from 2023 onwards under the new EU directive that has been recently adopted [6]. Predictive models can be useful in the future with regards to newly emerging DBPs. This study was limited by the small sample size when considering statistical modeling. Nevertheless, our approach would need to be validated to see whether the experimental data fits well with the predicted data in a larger set of samples. Finally, further research is needed in other settings to evaluate the site-specificity of the predictive models.

### Effect of domestic filters on DBPs concentrations in tap water

Our findings showed that domestic activated carbon and reverse osmosis filters, in real operating conditions in the general population, removed DBPs from tap water to a variable extent. Activated carbon filters reduced DBP concentrations in the range of 27–80% depending on the class. Previous studies showed that activated carbon filters were able to remove DBPs by ~97% [46–48]. Our study was conducted in real operating conditions, and the carbon filters were not likely in optimal state of maintenance. Activated carbon has a limited useful life, and as they filter the water they accumulate compounds until they become saturated. It is very important that the manufacturer’s instructions are followed and changed frequently. Reverse osmosis filters reduced DBP concentrations in the range of 98–100%, which is consistent with previous studies showing reverse osmosis to be the most efficient method in removing all types of contaminants including DBPs from water sources up to 99% [36, 47, 48]. However, it is important to note that reverse osmosis also remove minerals from drinking water, that may counteract the health benefits of DBP removal considering certain populations or geographical regions [48]. Confirmation of our findings in a larger set of samples is warranted.

### Urinary biomarkers of exposure

Our findings showed that TCAA was the most prevalent HAA in urine (69.2% >LOD; non-adjusted >LOD: mean = 4.2 μg/L, median = 1.3 μg/L; creatinine adjusted >LOD: mean = 3.0 μg/g, median = 1.3 μg/g. Comparable levels of mean urinary unadjusted TCAA concentrations were observed in the US general population sample (3.3 µg/L) [49] and in a sample of Chinese pregnant women (2.7 µg/L) [50]. However, higher concentrations have been reported in a UK sample of pregnant women (unadjusted mean = 6.1 µg/L) [13].

Although the use of biomarkers to estimate exposure for etiologically relevant periods is hampered by the short half-life of DBPs, urinary TCAA has been used as a proxy DBP biomarker [12, 13, 51, 52] given that half-life (2.1–6.3 days) is longer than consecutive exposure events. Due to its nonvolatile nature, urinary TCAA can potentially inform about the ingested DBP exposure. In this study, we evaluated the relationship between urinary TCAA and ingested TCAA calculated by self-reported at-home drinking water consumption questionnaire resulting in a statistically significant moderate (*r* = 0.48) correlation. This finding is directly in line with Smith et al. [13] that showed a significant moderate correlation (*r* = 0.50, *p* value=0.002) between ingested TCAA from home tap water and TCAA in urine as well as by Zhang et al. [12] showing a significant strong correlation (*r* = 0.66, *p* value < 0.001). On the contrary, some other studies did not report statistically significant correlations between urine and ingested TCAA from drinking water [10, 11, 49, 53]. All previous studies highlighted that the assessment of water consumption is the basis of measuring TCAA exposure variability whereas individual volume of tap water, source, behavioral differences and employment status are key factors that explain this variability. It is important to note that we collected drinking water samples at home, which does not reflect the total personal exposure to TCAA from drinking water. We acknowledge that it is a limitation as we estimated only part of the total personal exposure levels. However, at the time of sample collection the working practices shifted towards working remotely due to the COVID-19 pandemic. It is possible that this change may improve aspects of the exposure assessment regarding a better characterization of drinking water consumption for subjects working from home that may be reflected by the significant positive correlation between urinary and ingested TCAA tap water similar to the findings of Smith et al. [13]. Taken together, these results suggest that TCAA ingestion from home tap water can be a valid proxy for TCAA average exposure when self-reported water intake is accurately characterized.

In addition to TCAA, we detected 4 other HAAs in urine including DCAA (mean = 154.7 µg/g creatinine, min=0.3 µg/g creatinine, max = 549.6 µg/g creatinine) and DBCAA (mean = 113.0 µg/g creatinine, min=0.6 µg/g creatinine, max = 240.9 µg/g creatinine) that showed rather high mean concentrations in a few (<25%) samples. As isotopic dilution is the most reliable and robust method for mass spectrometric analysis, our quantification analysis of all HAAs has been carried out based on this method, but only the labeled ^13^C-TCAA standard was available. Two previous studies assessed HAAs in urine, which already indicate their great matrix effect when studying these compounds in urine and that focused only on TCA analysis [22, 54].

To our knowledge, this is the first work to determine occurrence of several HAAs in urine in a context of drinking water exposure. On the other hand, the use of social networks to recruit participants probably introduced selection bias and the study population may not be representative of the general adult population, although it would not affect internal validity. In addition, self-collected data of water consumption may have introduced measurement error to some extent in the estimation of the amount of water consumed. Finally, the use of a spot urine sample assessed cross sectionally with the water sample may partly explain the moderate correlation since these samples do not reflect the same exposure period.

## Conclusion

This study provides a broad description of DBPs occurrence in different types of drinking water and in urine specimens. DBP levels were below the recently established parametric values of the EU Drinking Water Directive (2020/2184) that will regulate a wider range of DBPs from 2023 onwards. Findings suggest that specific non-regulated DBPs can be predicted using linear regression models and machine learning algorithms based on routine monitoring data. Future investigations are needed to validate these predictive models in different settings. DBPs in tap water were partially to totally removed by domestic activated carbon and reverse osmosis filters. TCAA ingestion from home tap water explained ≈50% of urinary TCAA total variability, suggesting that TCAA ingestion from tap water can only partly explain urinary TCAA levels. Overall, these findings provide valuable insights for exposure assessment purposes in epidemiological studies.

### Supplementary information

Reporting Checklist

Supporting Information

## Data Availability

The data generated and analyzed during this study can be find within this published article and supplementary files.
