# Supplementary material for: Insights to estimate exposure to regulated and non-regulated disinfection by-products in drinking water
Source: J Expo Sci Environ Epidemiol. 2022 Jun 29;34(1):23–33. doi: 10.1038/s41370-022-00453-6 (PMC9244125; doi:10.1038/s41370-022-00453-6)
Supplement: Supplementary file 2 — Supporting Information [file 41370_2022_453_MOESM2_ESM.docx]

Supporting Information

Insights to estimate exposure to regulated and non-regulated disinfection by-products in drinking water

*Paula E. Redondo-Hasselerharm,*^†,^*^1,2,3^ Dora Cserbik,*^†,^*^1,2,3^ Cintia Flores,^4^ Maria J. Farré,^5,6^ Josep Sanchís, ^5,6^ Jose A. Alcolea,^1,2,3^ Carles Planas,^4^ Josep Caixach,^4^ Cristina M. Villanueva.^1,2,3,7,*^*

*^1^* ISGlobal, Barcelona, Spain

*^2^* Universitat Pompeu Fabra (UPF), Barcelona, Spain

*^3^* CIBER Epidemiología y Salud Pública (CIBERESP), Madrid, Spain

*^4^* Mass Spectrometry Laboratory/Organic Pollutants, Institute of Environmental Assessment and Water Research, IDAEA-CSIC, Barcelona, Spain

*^5^* Catalan Institute for Water Research, ICRA, Girona, Spain

^6^ University of Girona, Girona, Spain

*^7^* IMIM (Hospital del Mar Medical Research Institute), Barcelona, Spain

^†^ Equal contribution

^*^ Corresponding author:

Cristina Villanueva

ISGlobal - Institut de Salut Global de Barcelona

PRBB – Barcelona Biomedical Research Park

Doctor Aiguader, 88

08003 Barcelona, Spain

Tel. +34 93 214 73 44

cristina.villanueva@isglobal.org

**1. Analytical procedure.**

**1.1. Haloacetic acids in water.** We used on-line solid phase extraction (SPE) and tandem mass spectrometry coupled to liquid chromatography (LC-MS/MS) analysis. The internal standard 2,3-dibromopropanoic acid (DBPA) was added prior to analysis. A 1 mL aliquot was directly processed using a Thermo Electron’s EQuan environmental quantitation system. The system consists of two Surveyor LC and MS pumps with a preconcentration column, an analytical column, a PAL autosampler (CTC Analytics, Zwingen, Switzerland) and one switching device unit. The entire system was connected to a TSQ quantum triple quadrupole mass spectrometer. The SPE columns used were a combination of mixed-mode Strata-X cartridge (2.0×20 mm, 25 μm particle size; Phenomenex, Torrance, CA, USA) and plus Hypersil GOLD C18 (2.1×20 mm, 12 μm particle size; Thermo Fisher Scientific, Franklin, MA, USA). The pre-concentration was carried out at 1 mL/min with solvent A and B (95:5). After enrichment, analytes were transferred to the analytical column for their separation by switching the MS valve into loading mode. Luna C18 (2) column (150 × 2 mm, 5 μm) from Phenomenex (Torrance, CA, USA) was used for chromatographic separation and the mobile phase was composed of solvent A (0.1% formic acid in water) and solvent B (0.1% formic acid in acetonitrile) at a flow rate of 200 µL/min using a linear gradient. The total run time was 25 min.

**1.2. Chlorite and chlorate.** We used direct injection onto LC–MS/MS system. Chromatographic separation was performed by linear gradient elution on a reversed-phase Kinetex XB-C18 analytical column (100 × 2.1 mm, 2.6 µm) from (Phenomenex, Torrance, CA, USA) preceded by an ultra-high performance liquid chromatography (UHPLC) C18 Security Guard Ultra cartridge. The mobile phase was composed of water as solvent A and methanol as solvent B, both containing 0.1% (v/v) formic acid at a flow rate of 300 µL/min using a linear gradient. The total run time was 25 min. The sample volume injection was 50 µL.

**1.3.** **Trihalomethanes, haloacetonitriles, haloketones and trichloronitromethane*.*** Analysis was performed by liquid-liquid salted microextraction and gas chromatography (GC Trace 1300, Thermo Fisher Scientific) coupled to TSQ 9000 triple quadrupole mass spectrometer (GC–MS/MS) (Thermo Fisher Scientific) according to U.S. Environmental Protection Agency (US EPA) method 551.1 (U.S.EPA., 1995). The system also consists of an autosampler TriPlus RSH (Thermo Fisher Scientific). Sample pH was adjusted to pH 3.5 with 0.2 N sulphuric acid and extracted using 3 mL of MtBE containing 200 µg/L of d_6_-1,2-dibromopropane as internal standard. After the addition of ~10 g of high purity sodium sulphate, the samples were vortexed for 1 minute and left to settle for 5 min. Finally, ~1.5 mL of MtBE extract was transferred into 2 mL vials for injection. The injector was operated in splitless mode. Chromatographic separation was performed using a mid-polar column, a TraceGOLD TG-1701MS from Thermo Fisher Scientific (30 m × 0.25 mm × 0.5 µm). The oven temperature program was as follows: 35ºC for 5 min, ramp to 100ºC at 10ºC/min and then ramp to 200ºC at 20ºC/min and hold for 1 min. The inlet temperature was set at 200ºC. Acquired data was processed by TraceFinder 4.1 software.

**1.4. Physicochemical parameters*.*** Free and total chlorine were determined using a colorimetric test kit (Hanna instruments, Spain), with a resolution of 0.02 mg/L and an upper range of 5 mg/L. ﻿Conductivity was quantified with a conductometer (Hanna instruments, Spain) with a resolution of 1µS/cm and an upper range of 1999 µS/cm. pH was measured with a GLP 21 pHmeter (Crison Instruments, Spain). Total hardness was measured as CaCO_3_ using EDTA titration, with a resolution of 0.03 mg/L and an upper range of 500 mg/L (Hanna instruments, Spain). Total organic carbon (TOC) was assessed by 680°C combustion catalytic oxidation with a resolution of 0.05 mg C/L and a maximum range of 1000 mg C/L (Shimadzu, Spain).

**1.5. Haloacetic acids in urine.** As extraction internal standard, 50 µL of labeled trichloroacetic acid (^13^C-TCAA) 10 ng/µL was added to 3.5 mL of urine samples. Each sample was sonicated (20 min) and was centrifuged (3000 rpm, 10 min) to eliminate solid residue. The supernatant was diluted with 7 mL of water and 4.6 µL of formic acid. Oasis-HLB SPE cartridge, 200 mg/6 mL (Waters Corporation, Milford, MA), was conditioned with 5 mL of methanol and 5 mL of acidified water (0.1% formic acid). Then, the diluted urine was loaded onto the conditioned SPE cartridge. Finally, HAAs were eluted twice with 5 mL of MeOH:H_2_O 20:80. The extracts were evaporated to dryness and reconstituted with 150 µL of acidified MeOH:H_2_O 70:30 (0.1% HCOOH) and 50 µL of DBPA 10 ng/µL as injection internal standard. Prepared samples were stored at -20°C before analysis. The chromatographic separation was performed on a reversed phase Kinetex XB-C_18_ column (100 × 2.1 mm, 2.6 μm) preceded by an C_18_ guard column (2 × 2.1 mm, 2.6 μm) both from Phenomenex (Torrance, CA, USA) inside an oven at 40°C. The mobile phase was composed of solvent A (0.1% formic acid in water) and solvent B (0.1% formic acid in acetonitrile) using a linear gradient. The mobile phase flow rate was 200 µL/min. The total duration of the method was 25 min. The sample volume injection was 10 μL.

**1.6. Reagents.** All reagents were of liquid chromatographic (LC) grade. For all LC–MS/MS analyses, nitrogen (99.999% pure) and high-purity argon (Ar1) supplied by Air Liquide (Madrid, Spain) was used for the electrospray ionization (ESI) source and as a collision-induced dissociation (CID) gas in the triple quadrupole, respectively. For GC–MS analysis, helium (He2) obtained by Air Liquide (Madrid, Spain) was employed as carrier gas. Reagent water and formic acid were purchased from Merck (Darmstadt, Germany).

***1.6.1 Haloacetic acids, chlorate and chlorite*.** Ascorbic acid to quench chlorine in the bottles was purchased from Panreac (Castellar del Vallès, Spain). Reagent water, acetonitrile, methanol and formic acid were purchased from Merck (Darmstadt, Germany). A mix of HAA9 (monochloroacetic acid (MCAA), dichloroacetic acid, trichloroacetic acid (TCAA), monobromoacetic acid (MBAA), dibromoacetic acid (DBAA), tribromoacetic acid (TBAA), bromochloroacetic acid (BCAA), bromodichloroacetic (BDAA) acid and dibromoachloroacetic acid (DBCAA)) in MtBE at individual concentrations of 2 μg/μL, monoiodoacetic acid (MIAA), ^13^C-TCAA and DBPA, used as internal standards, were acquired from Sigma-Aldrich, Steinheim, Germany. Diiodoacetic acid (DIAA) was obtained from Toronto Research Chemicals (Toronto, Ontario, Canada). Chlorate and chlorite salts were purchased from Acros (Geel, Belgium).

***1.6.2.*** **Trihalomethanes, haloacetonitriles, haloketones and trichloronitromethane*.*** Ascorbic acid to quench chlorine was purchased from Sigma-Aldrich (Steinheim, Germany). Volatile trihalomethanes (THMs), haloacetonitriles (HANs), haloketones (HKs) and trichloronitromethane (TCNM) standards were obtained as a mix at 5.0 mg/mL in acetone (>95 % purity) from Cluzeau (Sainte-Foy-la-Grande, France). THMs were purchased as a 1.0 mg/mL mix in methanol (TraceCERT® grade) from Sigma-Aldrich, Steinheim, Germany. Deuterated 1,2-dibromopropane-d6 (99.6 atom % D) was purchased from CDN isotopes (Quebec, Canada) and used as internal standard. Ultrapure water, methanol (Optima® LC/MS grade), methyl tert-butyl ether (MTBE) (Chromasolv™ Plus), and anhydrous Na2SO3 (≥98.0 %, BioUltra grade) were purchased from Fisher Chemical (Geel, Belgium), Fisher Chemical, (Loughborough, United Kingdom), Merck (Darmstadt, Germany) and Sigma-Aldrich (Steinheim, Germany), respectively.

**2. Quantification and quality control**

According to the 2002/657/EC Commission Decision (European Commission, 2002), for each compound two MRM transitions by LC–MS/MS or two ions by GC–MS were monitored, one for quantification and one for confirmation. A relation between transitions or ions was calculated. For identification purposes, the following criteria were accomplished: i) the ratio of the chromatographic retention time of the analyte to that of the internal standard, shall correspond to that of the calibration solution at a tolerance of ±2.5%; ii) two m/z transitions were confirmed for each analyte; iii) the ratio between the two transitions in the sample compared to ratio in the calibration curve should be in agreement to [calibration curve average ± maximum permitted tolerances for relative ion intensities as proposed in 2002/657/EC Decision]. The quantification of HAAs was performed on the basis of the isotope dilution method. Therefore, internal standards, ^13^C-TCAA and DBPA, have been added to each analysis. The quantification of HAAs was performed by internal calibration curve: the plot ratio of the most intensive transition peak area divided by the internal standard area against the ratio of concentrations. THMs, HANs, HKs and HNMs were also quantified with internal calibration curve. Moreover, the calibration curve was extracted at the same time of the samples. On the other hand, chlorate and chlorite quantification was performed by external calibration.

For urine analysis, ^13^C-TCAA and DBPA were used as extraction and injection internal standards. The DBPA was used in order to calculate the recovery of ^13^C-TCA in each sample and check LC–MS/MS injection. Two different types of calibration curve were studied: matrix-matched calibration and solvent calibration. Due to the fact that an important matrix effect has been observed, samples were quantified by matrix-matched calibration. Additionally, in the optimization of the analytical methodology, thermal instability has been observed for the analysis of TBAA.

**3. Bromine incorporation factor**

The bromine incorporation factor (BIF) for THMs was calculated as follows, where concentrations are on a molar basis (Gould *et al*., 1983; Symons *et al*., 1993):

$$BIF(THMs)=\frac{0\times\left[ TCM \right]+1\times\left[ BDCM \right]+2\times\left[ DBCM \right]+3\times\left[ TBM \right]}{\left[ TCM \right]+ \left[ BDCM \right]+\left[ DBCM \right]+ \left[ TBM \right]}$$

(1)

For HAAs, BIF values were calculated for the trihalogenated (TXAAs) and dihalogenated species (DXAAs) using the following formulas, where concentrations are on a molar basis (Krasner *et al*., 2008):

$$BIF(DXAA)=\frac{0\times\left[ DCAA \right]+1\times\left[ BCAA \right]+2\times\left[ DBAA \right]}{\left[ DCAA \right]+\left[ BCAA \right]+\left[ DBAA \right]}$$

(2)

$$BIF(TXAA)=\frac{0\times\left[ TCAA \right]+1\times\left[ BDCAA \right]+2 \times\left[ DBCAA \right]+ 3\times\left[ TBAA \right]}{\left[ TCAA \right]+\left[ BDCAA \right]+\left[ DBCAA \right]+\left[ TBAA \right]}$$

(3)

In order to compare the calculated BIFs with those reported by Goslan *et al*. (2014), these were normalized by dividing them by the number of halogens. Thus, the BIF-THM and BIF-TXAA were divided by 3, while the BIF-DXAA was divided by 2. A value of 1 would indicate that only brominated species are present and a value of 0 would indicate the presence of chlorinated species only.

**Table S1.** Limits of quantification or detection (LOQ, LOD) and analytical technique for the disinfection by-products (DBPs) analyzed in water and urine samples.

|  | **LOQ in water (µg/L)** | **LOD in urine (µg/L)** | **Analytical technique** |
| --- | --- | --- | --- |
| **Haloacetic acids (HAAs)** |  |  | On-line SPE  LC–MS/MS |
| Monochloroacetic acid (MCAA) | 2.0 | 1.3 |  |
| Dichloroacetic acid (DCAA) | 0.5 | 0.7 |  |
| Trichloroacetic acid (TCAA) | 0.5 | 0.02 |  |
| Monobromoacetic acid (MBAA) | 0.5 | 0.05 |  |
| Dibromoacetic acid (DBAA) | 0.5 | 0.05 |  |
| Tribromoacetic acid (TBAA) | 0.5 | - |  |
| Bromochloroacetic acid (BCAA) | 0.5 | - |  |
| Bromodichloroacetic acid (BDCAA) | 0.5 | 0.06 |  |
| Dibromochloroaceti acid (DBCAA) | 0.5 | 1.6 |  |
| Iodoacetic acid (MIAA) | 0.5 | 3.98 |  |
| Diiodoacetic acid (DIAA) | 0.5 | 0.56 |  |
| **Trihalomethanes (THMs)** |  |  | LLE  GC–MS/MS |
| Chloroform (TCM) | 0.1 | **-** |  |
| Bromodichloromethane (BDCM) | 0.1 | **-** |  |
| Dibromochloromethane (DBCM) | 0.1 | **-** |  |
| Bromoform (TBM) | 0.1 | **-** |  |
| **Haloacetonitriles (HANs)** |  |  |  |
| Dichloroacetonitrile (DCAN) | 0.1 | **-** |  |
| Trichloroacetonitrile (TCAN) | 0.1 | **-** |  |
| Bromochloroacetonitrile (BCAN) | 0.1 | **-** |  |
| Dibromoacetonitrile (DBAN) | 0.1 | **-** |  |
| **Haloketones (HKs)** |  |  |  |
| 1,1-Dichloropropanone (DCP) | 0.1 | **-** |  |
| 1,1,1-Trichloropropanone (TCP) | 0.1 | **-** |  |
| **Other DBPs** |  |  |  |
| Trichloronitromethane (TCNM) | 0.1 | **-** |  |
| Chlorate | 10 | **-** | Direct injection  LC–MS/MS |
| Chlorite | 10 | **-** |  |

Note: The limit of quantification (LOQ) was considered the first level of the calibration curve. The limit of detection (LOD) was estimated as the concentration level that gives rise to a signal/noise ratio equal to three. No results were found in water samples with concentration levels <LOQ and >LOD, so we have considered the LOQ for water analysis. In contrast, for urine samples, the LOD has been taken as a reference.

**Table S2.** Descriptive statistics of the physicochemical parameters in tap water samples and bottled water samples.

|  | **N** | | **Min** | **Perc25** | **Perc50** | | **Perc75** | **Max** | **Mean** | **SD** | |
| --- | --- | --- | --- | --- | --- | --- | --- | --- | --- | --- | --- |
| **Tap water** | | | | | | | | | | | |
| Conductivity (µS/cm) | 42 | | 353 | 373 | 833 | | 941 | 1185 | 710 | 282 | |
| Hardness (mg/L) | 42 | | 120 | 172 | 249 | | 287 | 310 | 230 | 59.8 | |
| Total organic carbon (mg/L) | 42 | | 1.0 | 1.2 | 1.3 | | 1.9 | 2.3 | 1.5 | 0.4 | |
| pH | 42 | | 6.2 | 7.4 | 7.6 | | 7.8 | 8.0 | 7.5 | 0.4 | |
| Free chlorine (mg/L) | 23* | | 0.0 | 1.2 | 1.6 | | 1.8 | 2.4 | 1.5 | 0.6 | |
| Total chlorine (mg/L) | 23* | | 0.1 | 1.9 | 2.1 | | 2.4 | 2.7 | 2.2 | 0.5 | |
| **Bottled water** | | | | | | | | | | | |
| Conductivity (µS/cm) | 10 | 32.0 | | 186 | 230 | 324 | | 422 | 244 | 122 |  |
| Hardness (mg/L) | 10 | 33.0 | | 106 | 129 | 209 | | 276 | 147 | 79.9 |  |
| Total organic carbon (mg/L) | 10 | 0.2 | | 0.3 | 0.4 | 0.4 | | 0.7 | 0.4 | 0.1 |  |
| pH | 10 | 7.0 | | 7.3 | 7.4 | 7.6 | | 7.7 | 7.4 | 0.2 |  |
| Free chlorine (mg/L) | 10 | 0 | | <0.02 | <0.02 | <0.02 | | <0.02 | <0.02 | <0.02 |  |
| Total chlorine (mg/L) | 10 | 0 | | <0.02 | <0.02 | <0.02 | | <0.02 | <0.02 | <0.02 |  |

^(*)^ The number of analysed samples is reduced because a technical problem in part of the samples.

**Table S3.** Ratios between chloroform (TCM) and bromoform (TBM), and total trihalomethane (THM) and haloacetic acid (HAA) concentrations (µg/L) in the N=42 unfiltered tap water samples.

| **Sample number** | **TCM** | **TBM** | **Ratio** | **Total THMs** | **Total HAAs** |
| --- | --- | --- | --- | --- | --- |
| 1 | 0.6 | 30 | TBM > TCM | 42 | 2.7 |
| 2 | <LOQ | 24 | TBM > TCM | 35 | 20 |
| 3 | 0.9 | 26 | TBM > TCM | 34 | 4.5 |
| 4 | <LOQ | 35 | TBM > TCM | 48 | 11 |
| 5 | 3.2 | 41 | TBM > TCM | 60 | 3.0 |
| 6 | 25 | 14 | TCM > TBM | 53 | 38 |
| 7 | <LOQ | 27 | TBM > TCM | 36 | 10 |
| 8 | <LOQ | 25 | TBM > TCM | 34 | 15 |
| 9 | <LOQ | 26 | TBM > TCM | 39 | 12 |
| 10 | <LOQ | 45 | TBM > TCM | 64 | 6.7 |
| 11 | <LOQ | 23 | TBM > TCM | 35 | 14 |
| 12 | 2.30 | 28 | TBM > TCM | 41 | 4.3 |
| 13 | 30 | 3.2 | TCM > TBM | 50 | 38 |
| 14 | <LOQ | 27 | TBM > TCM | 37 | 18 |
| 15 | 0.3 | 20 | TBM > TCM | 26 | 4.6 |
| 16 | 28 | 0.4 | TCM > TBM | 41 | 37 |
| 17 | 13 | 25 | TBM > TCM | 57 | 17 |
| 18 | 0.4 | 21 | TBM > TCM | 28 | <LOQ |
| 19 | 1.5 | 32 | TBM > TCM | 48 | 20 |
| 20 | <LOQ | 33 | TBM > TCM | 49 | 11 |
| 21 | 28 | 0.6 | TCM > TBM | 40 | 38 |
| 22 | 27 | 2.3 | TCM > TBM | 40 | 39 |
| 23 | 26 | 3.6 | TCM > TBM | 42 | 30 |
| 24 | 30 | 3.6 | TCM > TBM | 46 | 21 |
| 25 | 36 | 0.2 | TCM > TBM | 48 | 36 |
| 26 | 27 | <LOQ | TCM > TBM | 38 | 34 |
| 27 | 24 | 11 | TCM > TBM | 52 | 38 |
| 28 | <LOQ | 52 | TBM > TCM | 82 | 14 |
| 29 | <LOQ | 27 | TBM > TCM | 39 | 16 |
| 30 | 0.8 | 24 | TBM > TCM | 46 | 18 |
| 31 | 34 | 1.3 | TCM > TBM | 47 | 36 |
| 32 | 36 | <LOQ | TCM > TBM | 47 | 39 |
| 33 | 15 | 21 | TBM > TCM | 55 | 28 |
| 34 | <LOQ | 27 | TBM > TCM | 39 | 7.1 |
| 35 | 23 | <LOQ | TCM > TBM | 33 | 24 |
| 36 | 0.6 | 23 | TBM > TCM | 31 | 5.0 |
| 37 | <LOQ | 58 | TBM > TCM | 83 | 16 |
| 38 | 0.6 | 13 | TBM > TCM | 17 | 3.1 |
| 39 | <LOQ | 45 | TBM > TCM | 65 | 26 |
| 40 | <LOQ | 29 | TBM > TCM | 38 | 14 |
| 41 | 21 | 10 | TCM > TBM | 45 | 25 |
| 42 | 35 | <LOQ | TCM > TBM | 48 | 30 |

**Table S4.** Principal component analysis of the disinfection by-products (N=42).

| **Component** | **PC1** | | **PC2** | |
| --- | --- | --- | --- | --- |
| Total variance explained | **61.5%** | | **16.3%** | |
|  | **Contribution to the component (%)** | **Coordinates** | **Contribution to the component (%)** | **Coordinates** |
| Dichloroacetic acid (DCAA) | 7.7 | 0.92 | 1.7 | -0.22 |
| Trichloroacetic acid (TCAA) | 8.4 | 0.96 | 0.7 | -0.14 |
| Monobromoacetic acid (MBAA) | 4.5 | -0.71 | 1.1 | -0.18 |
| Dibromoacetic acid (DBAA) | 5.5 | -0.78 | 5.6 | -0.41 |
| Tribromoacetic acid (TBAA) | 3.4 | -0.61 | 10.1 | -0.54 |
| Bromochloroacetic acid (BCAA) | 1.8 | 0.45 | 8.6 | -0.50 |
| Bromodichloroacetic acid (BDCAA) | 6.9 | 0.88 | 2.4 | -0.26 |
| Dibromochloroaceti acid (DBCAA) | 2.6 | -0.53 | 13.0 | -0.62 |
| Chloroform (TCM) | 8.3 | 0.96 | 0.7 | -0.15 |
| Bromodichloromethane (BDCM) | 7.4 | 0.91 | 3.2 | -0.31 |
| Dibromochloromethane (DBCM) | 5.1 | -0.75 | 7.3 | -0.46 |
| Bromoform (TBM) | 7.3 | -0.90 | 1.1 | -0.18 |
| Dichloroacetonitrile (DCAN) | 8.5 | 0.97 | 0.8 | -0.16 |
| Bromochloroacetonitrile (BCAN) | 0.01 | -0.03 | 23.7 | -0.83 |
| Dibromoacetonitrile (DBAN) | 8.0 | -0.94 | 1.3 | -0.19 |
| 1,1,1-Trichloropropanone (TCP) | 7.4 | 0.90 | 0.8 | -0.15 |
| Chlorate | 0.2 | 0.14 | 15.6 | -0.68 |
| Chlorite | 7.0 | 0.88 | 2.3 | -0.26 |

**Table S5.** Spearman correlation coefficients between disinfection by-products (DBPs), N=42 (values <LOQ were imputed LOQ/2). Bold indicates p-value<0.05.

|  | **Trihalomethanes (THMs)** | | | | | **Haloactic acids (HAAs)** | | | | | | | | | **Haloacetonitriles (HANs)** | | | | **Other DBPs** | |
| --- | --- | --- | --- | --- | --- | --- | --- | --- | --- | --- | --- | --- | --- | --- | --- | --- | --- | --- | --- | --- |
|  | TCM | BDCM | DBCM | TBM | Total THM | DCAA | TCAA | MBAA | DBAA | TBAA | BCAA | BDCAA | DBCAA | Total HAA | DCAN | BCAN | DBAN | Total HAN | TCP | Chlorite |
| BDCM | **0.79** |  |  |  |  |  |  |  |  |  |  |  |  |  |  |  |  |  |  |  |
| DBCM | **-0.70** | **-0.35** |  |  |  |  |  |  |  |  |  |  |  |  |  |  |  |  |  |  |
| TBM | **-0.80** | **-0.57** | **0.88** |  |  |  |  |  |  |  |  |  |  |  |  |  |  |  |  |  |
| Total THM | 0.17 | **-0.86** | **0.56** | **0.76** |  |  |  |  |  |  |  |  |  |  |  |  |  |  |  |  |
| DCAA | **0.87** | **0.84** | **-0.66** | **-0.81** | 0.23 |  |  |  |  |  |  |  |  |  |  |  |  |  |  |  |
| TCAA | **0.91** | **0.85** | **-0.63** | **-0.74** | 0.27 | **0.91** |  |  |  |  |  |  |  |  |  |  |  |  |  |  |
| MBAA | **-0.61** | **-0.47** | **0.73** | **0.70** | 0.13 | **-0.63** | **-0.60** |  |  |  |  |  |  |  |  |  |  |  |  |  |
| DBAA | **-0.78** | **-0.49** | **0.72** | **0.68** | 0.06 | **-0.66** | **-0.70** | **0.67** |  |  |  |  |  |  |  |  |  |  |  |  |
| TBAA | **-0.60** | -0.28 | **0.61** | **0.53** | 0.10 | **-0.48** | **-0.49** | **0.42** | **0.73** |  |  |  |  |  |  |  |  |  |  |  |
| BCAA | **0.35** | **0.41** | -0.12 | **-0.30** | 0.26 | **0.47** | **0.43** | -0.01 | 0.01 | 0.01 |  |  |  |  |  |  |  |  |  |  |
| BDCAA | **0.77** | **0.78** | **-0.61** | **-0.74** | 0.21 | **0.90** | **0.85** | **-0.60** | **-0.58** | **-0.36** | **0.46** |  |  |  |  |  |  |  |  |  |
| DBCAA | **-0.52** | -0.20 | **0.61** | **0.47** | 0.16 | **-0.41** | **-0.42** | **0.40** | **0.69** | **0.97** | 0.06 | -0.29 |  |  |  |  |  |  |  |  |
| Total HAA | **0.66** | **0.82** | **-0.44** | **-0.64** | **0.32** | **0.85** | **0.79** | **-0.43** | -0.27 | -0.07 | **0.63** | **0.85** | -0.01 |  |  |  |  |  |  |  |
| DCAN | **0.87** | **0.81** | **-0.67** | **-0.77** | 0.15 | **0.89** | **0.91** | **-0.58** | **-0.70** | **-0.46** | **0.42** | **0.81** | **-0.40** | **0.75** |  |  |  |  |  |  |
| BCAN | 0.16 | **0.55** | **0.43** | 0.15 | **0.81** | 0.21 | 0.24 | 0.17 | 0.18 | 0.23 | 0.34 | 0.22 | 0.30 | **0.42** | 0.20 |  |  |  |  |  |
| DBAN | **-0.78** | **-0.53** | **0.91** | **0.93** | 0.26 | **-0.79** | **-0.73** | **0.76** | **0.74** | **0.59** | -0.22 | **-0.72** | **0.55** | **-0.56** | **-0.76** | 0.30 |  |  |  |  |
| Total HAN | **-0.41** | -0.03 | **0.78** | **0.68** | **0.59** | **-0.35** | -0.28 | **0.63** | **0.56** | **0.54** | 0.07 | **-0.31** | **0.53** | -0.08 | -0.26 | **0.69** | **0.79** |  |  |  |
| TCP | **0.76** | **0.76** | **-0.68** | **-0.72** | 0.12 | **0.84** | **0.85** | **-0.67** | **-0.62** | **-0.45** | 0.30 | **0.79** | **-0.41** | **0.75** | **0.83** | 0.11 | **-0.75** | **-0.36** |  |  |
| Chlorite | **0.59** | **0.71** | **-0.48** | **-0.55** | 0.27 | **0.78** | **0.69** | **-0.48** | **-0.39** | -0.24 | **0.49** | **0.77** | -0.20 | **0.79** | **0.71** | 0.26 | **-0.54** | -0.13 | **0.75** |  |
| Chlorate | 0.03 | **0.41** | 0.14 | 0.01 | **0.40** | 0.26 | 0.22 | -0.12 | 0.13 | 0.15 | 0.22 | **0.35** | 0.18 | **0.44** | 0.16 | **0.39** | 0.02 | 0.27 | **0.38** | **0.58** |

**Table S6.** Spearman correlation coefficients between physicochemical parameters and chemicals measured in tap water. Bold indicates p-value<0.05.

|  | **Conductivity** | **Hardness** | **TOC** | **pH** | **Free Chl** | **Total Chl** |
| --- | --- | --- | --- | --- | --- | --- |
| **Hardness** | **0.86** |  |  |  |  |  |
| **Total Organic Carbon (TOC)** | **-0.83** | **-0.74** |  |  |  |  |
| **pH** | -0.28 | **-0.32** | **0.41** |  |  |  |
| **Free Chlorine (Free Chl)** | -0.11 | -0.15 | -0.21 | -0.05 |  |  |
| **Total Chlorine (Total Chl)** | -0.10 | -0.02 | -0.05 | 0.10 | **0.87** |  |
| **Haloacetic acids (HAAs)** | | | | | | |
| Dichloroacetic acid (DCAA) | **-0.81** | **-0.76** | **0.86** | **0.50** | 0.04 | -0.02 |
| Trichloroacetic acid (TCAA) | **-0.83** | **-0.69** | **0.81** | **0.52** | -0.02 | -0.04 |
| Monobromoacetic acid (MBAA) | **0.50** | **0.38** | **-0.57** | **-0.50** | -0.05 | -0.22 |
| Dibromoacetic acid (DBAA) | **0.47** | **0.41** | **-0.53** | **-0.63** | 0.043 | 0.05 |
| Tribromoacetic acid (TBAA) | 0.22 | 0.12 | -0.28 | **-0.41** | 0.13 | 0.07 |
| Bromochloroacetic acid (BCAA) | **-0.42** | **-0.47** | **0.39** | 0.10 | **0.43** | 0.31 |
| Bromodichloroacetic acid (BDCAA) | **-0.77** | **-0.67** | **0.81** | **0.46** | -0.02 | -0.01 |
| Dibromochloroaceti acid (DBCAA) | 0.14 | 0.03 | -0.24 | **-0.37** | 0.22 | 0.12 |
| Total HAAs | **-0.82** | **-0.76** | **0.82** | 0.30 | 0.06 | -0.02 |
| BIF-DXAA | **0.66** | **0.64** | **-0.67** | **-0.58** | -0.05 | 0.05 |
| BIF-TXAA | **0.65** | **0.55** | **-0.65** | **-0.50** | 0.06 | 0.07 |
| **Trihalomethanes (THMs)** | | | | | | |
| Chloroform (TCM) | **-0.73** | **-0.66** | **0.67** | **0.54** | -0.02 | -0.11 |
| Bromodichloromethane (BDCM) | **-0.91** | **-0.79** | **0.82** | **0.36** | -0.01 | 0.03 |
| Dibromochloromethane (DBCM) | **0.39** | **0.36** | **-0.48** | **-0.57** | 0.06 | 0.13 |
| Bromoform (TBM) | **0.62** | **0.65** | **-0.58** | **-0.50** | -0.18 | 0.05 |
| Total THMs | **-0.39** | -0.24 | **0.35** | -0.09 | -0.18 | 0.00 |
| BIF-THM | **0.82** | **0.79** | **-0.73** | **-0.52** | -0.06 | 0.00 |
| **Other disinfection by-products (DBPs)** | | | | | | |
| Dichloroacetonitrile (DCAN) | **-0.78** | **-0.69** | **0.82** | **0.53** | 0.13 | 0.05 |
| Bromochloroacetonitrile (BCAN) | **-0.41** | 0.29 | 0.27 | -0.14 | 0.10 | 0.03 |
| Dibromoacetonitrile (DBAN) | **0.60** | **0.60** | **-0.64** | **-0.54** | -0.10 | -0.09 |
| Total HANs | 0.15 | 0.21 | -0.15 | **-0.42** | 0.04 | -0.05 |
| 1,1,1-Trichloropropanone (TCP) | **-0.75** | **-0.64** | **0.81** | **0.54** | -0.15 | 0.00 |
| Chlorate | **-0.48** | -0.27 | **0.42** | 0.04 | -0.03 | 0.19 |
| Chlorite | **-0.70** | **-0.58** | **0.78** | **0.48** | 0.03 | 0.18 |

**Table S7.** Descriptive statistics of the physicochemical parameters in tap water before and after the use of activated carbon (AC) and reverse osmosis (RO) filters.

|  | **N** | **Min** | **Perc25** | **Perc50** | **Perc75** | **Max** | **Mean** | **SD** |
| --- | --- | --- | --- | --- | --- | --- | --- | --- |
| **Conductivity (µS/cm)** | | | | | | | | |
| Before AC filter | 6 | 366 | 470 | 735 | 896 | 937 | 684 | 252 |
| After AC filter | 6 | 247 | 448 | 672 | 797 | 872 | 612 | 251 |
| Before RO filter | 5 | 364 | 823 | 941 | 951 | 984 | 813 | 258 |
| After RO filter | 5 | 16.0 | 61.0 | 64.0 | 95.0 | 101 | 67.4 | 33.9 |
| **Hardness (mg/L)** | | | | | | | | |
| Before AC filter | 6 | 168 | 183 | 231 | 254 | 255 | 219 | 41.0 |
| After AC filter | 6 | 30.0 | 60.0 | 153 | 230 | 246 | 145 | 97.1 |
| Before RO filter | 5 | 165 | 258 | 270 | 288 | 300 | 256 | 53.5 |
| After RO filter | 5 | 4.80 | 18.0 | 18.0 | 30.0 | 33.0 | 20.8 | 11.2 |
| **Total organic carbon (mg/L)** | | | | | | | | |
| Before AC filter | 6 | 1.2 | 1.3 | 1.4 | 1.9 | 2.3 | 1.6 | 0.5 |
| After AC filter | 6 | 1.1 | 1.2 | 1.2 | 1.7 | 2.1 | 1.5 | 0.4 |
| Before RO filter | 5 | 1.2 | 1.2 | 1.2 | 1.2 | 1.9 | 1.3 | 0.3 |
| After RO filter | 5 | 0.2 | 0.3 | 0.3 | 0.4 | 0.5 | 0.3 | 0.1 |
| **pH** | | | | | | | | |
| Before AC filter | 6 | 7.3 | 7.4 | 7.6 | 7.7 | 8.0 | 7.6 | 0.3 |
| After AC filter | 6 | 6.8 | 6.8 | 7.0 | 7.4 | 7.5 | 7.1 | 0.3 |
| Before RO filter | 5 | 7.5 | 7.6 | 7.6 | 7.6 | 7.8 | 7.6 | 0.1 |
| After RO filter | 5 | 6.8 | 7.4 | 7.5 | 7.7 | 7.8 | 7.4 | 0.4 |
| **Free chlorine (mg/L)*** | | | | | | | | |
| Before AC filter | 3 | 1.6 | 1.7 | 1.8 | 2.1 | 2.4 | 1.9 | 0.4 |
| After AC filter | 3 | 0 | 0.1 | 0.1 | 0.2 | 0.2 | 0.1 | 0.1 |
| Before RO filter | 4 | 0 | 1.0 | 1.4 | 1.5 | 1.7 | 1.1 | 0.8 |
| After RO filter | 4 | 0 | <0.02 | <0.02 | <0.02 | <0.02 | <0.02 | <0.02 |
| **Total chlorine (mg/L)^*^** | | | | | | | | |
| Before AC filter | 3 | 2.1 | 2.3 | 2.5 | 2.6 | 2.7 | 2.4 | 0.3 |
| After AC filter | 3 | 0.1 | 0.1 | 0.2 | 0.3 | 0.4 | 0.2 | 0.2 |
| Before RO filter | 4 | 0.1 | 1.5 | 2.0 | 2.2 | 2.4 | 1.6 | 1.0 |
| After RO filter | 4 | 0 | <0.02 | <0.02 | <0.02 | <0.02 | <0.02 | <0.02 |

^(*)^ The number of samples is reduced because a technical problem with part of the samples.


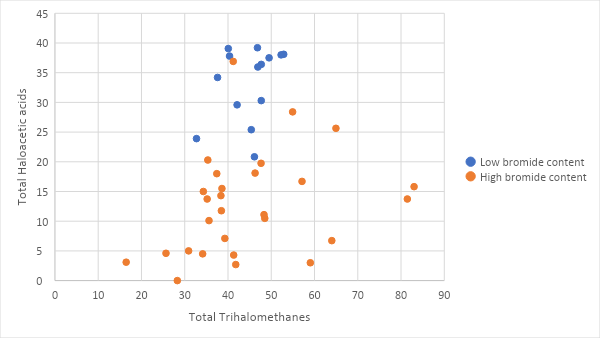


**Figure S1.** Concentrations (µg/L) of the sum of trihalomethanes (THMs) and the sum of haloacetic acids (HAAs) in the 42 samples collected in Barcelona, August-October 2020.

**
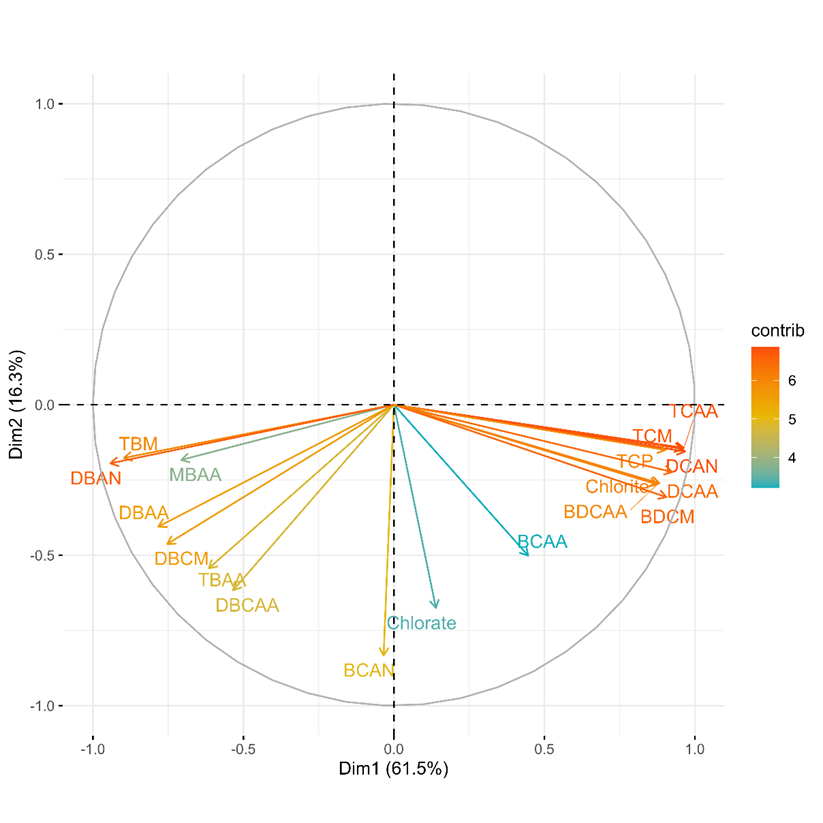
**

**Figure S2.** Principal component analysis with the disinfection by-products measured in tap water samples. Measurements <LOQ were assigned LOQ/2.

**References:**

European Commission, 2002. Comission decision of 12 August 2002 implementing Council Directive 96/23/EC concerning the performance of analytical methods and the interpretation of results (2002/657/EC), Official Journal of the European Communities.

Goslan, E.H., Krasner, S.W., Villanueva, C.M., Turigas, G.C., Toledano, M.B., Kogevinas, M., Stephanou, E.G., Cordier, S., Gražulevičiene, R., Parsons, S.A., Nieuwenhuijsen, M.J., 2014. Disinfection by-product occurrence in selected European waters. J. Water Supply Res. Technol. - AQUA 63, 379–390. <https://doi.org/10.2166/aqua.2013.017>

Gould, J., Fitchhorn, L., Urheim, E., 1983. Formation of brominated trihalomethanes: extent and kinetics, in: Jolley, R.L. (Ed.), Water Chlorination: Environmental Impact and Health Effects, Vol. 4. Ann Arbor Sci. Publ.

Krasner, S.W., Lee, C.F.T., Chinn, R., Hartono, S., Weinberg, H.S., Richardson, S.D., Pressman, J., Speth, T.F., Miltner, R., Simmons, J.E., 2008. Bromine incorporation in regulated and emerging DBPs and the relative predominance of mono-, di-, and trihalogenated DBPs. Proc. AWWA WQTC. Denver, Color. AWWA 1692–1708.

Symons, J.M., Krasner, S.W., Simms, L.A., Sclimenti, M., 1993. Measurement of THM and precursor concentrations revisited: the effect of bromide ion. J. / Am. Water Work. Assoc. 85, 51–62. <https://doi.org/10.1002/j.1551-8833.1993.tb05921.x>

U.S. EPA. (1995). Method 551.1: Determination of Chlorination Disinfection Byproducts, Chlorinated Solvents, and Halogenated Pesticides/Herbicides in Drinking Water by Liquid-Liquid Extraction and Gas Chromatography With Electron-Capture Detection. Revision 1.0. Cincinnati, OH
